# Prevalence of *Cryptosporidia, Eimeria*, *Giardia*, and *Strongyloides* in pre-weaned calves on smallholder dairy farms in Mukurwe-ini district, Kenya

**DOI:** 10.14202/vetworld.2015.1118-1125

**Published:** 2015-09-22

**Authors:** Getrude Shepelo Peter, George Karuoya Gitau, Charles Matiku Mulei, John Vanleeuwen, Shauna Richards, Jeff Wichtel, Fabienne Uehlinger, Omwando Mainga

**Affiliations:** 1 Department of Clinical Studies, Faculty of Veterinary Medicine, University of Nairobi, P.O. Box 29053-00625, Kangemi, Kenya; 2 Department of Health Management, Centre for Veterinary Epidemiological Research, Atlantic Veterinary College, University of Prince Edward Island, 550 University Avenue, Charlottetown PEI Canada, C1A 4P3, Canada; 3 Department of Large Animal Clinical Sciences, Western College of Veterinary Medicine, University of Saskatchewan, 52 Campus Drive, Saskatoon SK Canada, S7N 5B4, Canada; 4 Department of Public Health, Pharmacology, and Toxicology, Faculty of Veterinary Medicine, University of Nairobi, P.O. Box 29053-00625, Kangemi, Kenya

**Keywords:** *Cryptosporidia, Eimeria*, *Giardia*, pre-weaned calves, smallholder dairy farms, *Strongyloides*

## Abstract

**Aim:**

Gastrointestinal diseases are among the leading causes of calf morbidity and mortality in Kenya and elsewhere. This study was undertaken to determine the prevalence of *Cryptosporidia, Eimeria, Giardia*, and *Strongyloides* in calves on smallholder dairy farms (SDF) in Mukurwe-ini District, Nyeri County, Kenya. These infections have been associated with economic losses by decreased growth rates, decreased productivity, and increased susceptibility to other diseases.

**Materials and Methods:**

An observational study was conducted on 109 farms in Mukurwe-ini District, Nyeri County, Kenya, where 220 calf fecal samples (each calf at 4 and 6 weeks of age) from 110 calves (1 set of twins) were collected and analyzed for *Cryptosporidia, Eimeria, Giardia*, and helminth parasites.

**Results:**

*Eimeria* oocysts, *Cryptosporidia* oocysts, and *Strongyloides* eggs were detected in the fecal samples examined, but no *Giardia* cysts were found. The overall period prevalence of *Eimeria, Cryptosporidia*, and *Strongyloides* was 42.7% (47/110), 13.6% (15/110), and 5.4% (6/110), respectively. The prevalence at 4 weeks of age for *Eimeria, Cryptosporidia*, and *Strongyloides* was 30.0% (33/110), 8.2% (9/110), and 3.7% (4/109), respectively, while the prevalence at 6 weeks of age was 20.2% (22/109), 6.5% (7/107), and 2.7% (3/110), respectively. There was, however, no significant difference in the prevalence at 4 and 6 weeks (p>0.05).

**Conclusion:**

Findings from this study show that *Eimeria*, *Cryptosporidia*, and *Strongyloides*, are prevalent in the study area and indicate the need to adopt optimal management practices to control infections in calves.

## Introduction

Gastrointestinal (GI) and respiratory diseases are the leading causes of calf morbidity and mortality in Kenya and Elsewhere [1-3]. GI diseases in calves can be caused by protozoa such as *Cryptosporidium*, *Coccidia*, and *Giardia;* bacteria such as *Escherichia coli*; and viruses such as rotavirus and coronavirus [4,5]. *Cryptosporidia*, *Coccidia*, and *Giardia* have been associated with both clinical and sub-clinical disease in calves, resulting in great economic losses by decreased growth rates, decreased productivity, and increased susceptibility to other diseases [6-8]. GI nematode infections in cattle are widespread in the world [9], including herds in Kenya [8], causing constraints to productivity through decreased growth rates and mortalities.

*Cryptosporidia* is considered as one of the most common causes of neonatal diarrhea in dairy herds [10,11]. There are a number of species of *Cryptosporidium* that infect cattle, and these include *Cryptosporidium parvum, Cryptosporidium ryanae, Cryptosporidium andersoni*, and *Cryptosporidium hominis* [12]. Cryptosporidium *parvum* is the most common of the species, commonly found in young calves, and together with *C. hominis*, they have zoonotic potential. Young calves are therefore a source of infection to humans [13-15]. In Africa, the prevalence of *Cryptosporidia* among the pre-weaned dairy calves varied from 16.5% in Mogogoro Tanzania [16] and 18% in Kenya [17] to 34% in Zambia [18]. Bovine eimeriosis is mainly an infection of young animals [8,19]. Most *Coccidia* species are host specific, and the most pathogenic in cattle are *Eimeria bovis* and *Eimeria zuernii* [8,20,21]. In Kenya, a prevalence of 69.3% in calves has been reported by Waruiru *et al*. [8], and the following prevalences have been reported elsewhere in the developing world: 52% in South Africa [22], 48% in Argentina [23], 50% in India [24], and 51.8% in China [25]. Giardiasis is a common protozoal disease of cattle, especially calves [26]. *Giardia duodenalis* is the species reported to infect humans and a wide range of mammalian species [27]. *Giardia* *duodenalis* has seven assemblages which have host preferences, and assemblage E is known to infect livestock [6,28,29]. There is also evidence that the species that affect animals also affect human beings [30-32]. The greatest risk of zoonotic transmission seems to come from dogs and cats [33] and dairy cattle [34] who act as reservoirs. The major GI nematodes are *Haemonchus, Trichostrongylus, Cooperia, and Oesophagostomum* [35]. *Strongyloides* is a minor nematode species, but can cause sudden death without any clinical signs in young calves due to hyperinfection with larvae [36]. Pre-weaned calves typically only get exposed to *Strongyloides* prior to consuming grass. The prevalence of *Strongyloides* has been reported on smallholder farms in the following African countries: 7% in Ethiopia [37]; 4.0% in Kiambu District in Kenya [8]; and 2-5% in Nyandarua District in Kenya [38].

Prevention remains a very critical intervention in the management of calf diseases, especially of the GI tract (GIT). This study was therefore undertaken to determine the prevalence of *Cryptosporidia, Eimeria*, *Giardia*, and *Strongyloides* in order to guide efforts needed to control them through better management. Additionally, many calves in smallholder dairy farms (SDF) are hand-fed, making it important to survey the prevalence of *Cryptosporidia* and *Giardia* which have zoonotic potential [15].

## Materials and Methods

### Ethical approval

The study was approved by Biosecurity, Animal use and Ethics committee, Faculty of Veterinary Medicine, University of Nairobi. The farmers were recruited into the study, signed a consent form.

### Study area

The study was carried out in Mukurwe-ini District, Nyeri County, Kenya. Nyeri County is one of the 47 counties in Kenya, and one of the five counties of the former central province. It is part of Kenya’s Central Highlands. Nyeri County is located between longitude 36° and 38° east and between the equator and latitude 1° south. Mount Kenya is located to the east of Nyeri County at an altitude of 5199 m, and the Aberdare Range is to the west at 3999 m [39]. Nyeri County is part of Kenya’s Central Highlands and is sub-divided into seven Districts: Othaya, Mukurwe-ini, Mathira, Kieni East, Kieni West, Municipality, and Tetu. Mukurwe-ini District covers an area of 180.5 km^2^ with a population of 87,447 persons [40]. It is located 1644 m above sea level. The District has four divisions and is gazetted as; Mukurwe-ini West (Gakindu), Mukurwe-ini East (Giathugu and Rutune), Mukurwe-ini Central (Muhito and Githi), and Mukurwe-ini North (Gikondi and Thanu) [41].

The topography of the study area is characterized by steep ridges and valleys, especially the southern part of Mukurwe-ini. In addition to the fertile soils, the study area receives adequate equatorial rainfall, making it suitable for the crop (coffee and tea farming) and dairy farming. Dairy farming is mainly done on smallholder farms where zero-grazing is practiced. In these units, farmers cut fodder either from their farms or roadsides and take it to the cattle [42].

### Farm and calf selection and study design

Dairy farms used in this study were selected from Mukurwe-ini Wakulima Dairy Limited (MWDL), which has approximately 6,000 active members. Purposive sampling was used to select smallholder farms (i.e., having up to 4 cows) to be included in the study. Artificial insemination records kept by the dairy company were queried to identify farms with dams that were due to calf in the months of June and July 2013 (rainy season in Kenya). The farms whose records indicated that they had a cow that was to calve within the study period were contacted by phone and invited to participate in the study. In order to be included, farmers had to agree to keep the calf for at least the first 6 weeks of its life (the study period). When a farmer agreed to participate in the study, he/she was requested to contact MWDL as soon as the cow calved. A total of 109 farms were selected into the study with a total number of 110 calves (one farm had twins).

The farms were first visited within a week of the calf being born. Each of the study calves was allocated a study number and ear tagged for identification. Subsequent visits were made at 4 and 6 weeks of age for fecal sampling.

### Fecal sample collection

Two fecal samples were rectally collected from each calf; one at 4 weeks and the second one at 6 weeks of age. The samples were labeled and placed in fecal bottles and transported in a cool box to the laboratory for analysis. In the laboratory, analysis of *Eimeria, Cryptosporidia, Giardia* parasites, and helminth eggs was done as described in the next section.

### Fecal parasite analysis

#### Analysis of Cryptosporidia, Eimeria, Strongyloides, and Giardia

Sheather’s sucrose floatation method was used to harvest the *Cryptosporidium* oocysts, and slides were subsequently stained using modified Ziehl-Neelsen stain as described by Pfukenyi *et al*. [43]. On observation of the slide under a light microscope at ×100 oil immersion, the oocysts stained red with varying degrees of intensity against a green background, whereas the most fecal debris and yeast cells take up the color of the counterstain (Malachite green). One slide per sample was analyzed. The samples were considered positive if at least one morphologically distinct *Cryptosporidium* oocyst was observed (round to oval oocyst and at times a black dot or small vacuoles in oocyst could be seen).

The McMaster technique was used for determining the number of nematode eggs and *Eimeria* oocysts per gram of feces [44]. Both compartments of the McMaster counting chamber were filled with the sub-sample then allowed to stand for 5 min, before being examined under a light microscope (×100) and all eggs and *Eimeria* oocysts within the engraved area of the chamber were counted. The number of eggs per gram (EPG) was calculated as the number of eggs within the grid of each chamber multiplied by a factor of 50 [44]. *Strongyloides papillosus* eggs were identified by the characteristic sphere-shaped egg with a thin wall, flattened edges and with a larva inside. *Strongyloides* was reported when EPG was ≥200 since this was considered as moderate to high infection [45]. The presence of any coccidian oocysts was defined as infected.

The formol-ether parasite concentration technique was used to harvest *Giardia* cysts, as described by Cheesbrough [46]. The sediment content was placed on a microscope slide and examined under the light microscope at ×100 magnification. The presence of one *Giardia* cyst in a sample was reported as an infection.

### Data analysis

The laboratory results were manually entered into an excel spreadsheet [47]. The data were then imported to SPSS for windows, Version 20. Prevalence of the infections was made based on detection of *Cryptosporidia* and *Eimeria* oocysts or *Giardia* cysts in at least one of the examined fecal samples for each calf, and *Strongyloides* eggs (EPG ≥200). Chi-square was used to calculate the p-value to determine the difference in prevalence of the parasites at 4 and 6 weeks of age.

## Results

**Figure-1:**
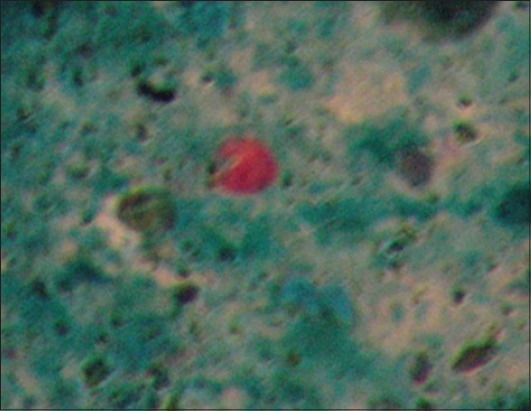
*Cryptosporidium* oocyst after staining with Modified Ziehl-Neelsen (×1000).

**Figure-2:**
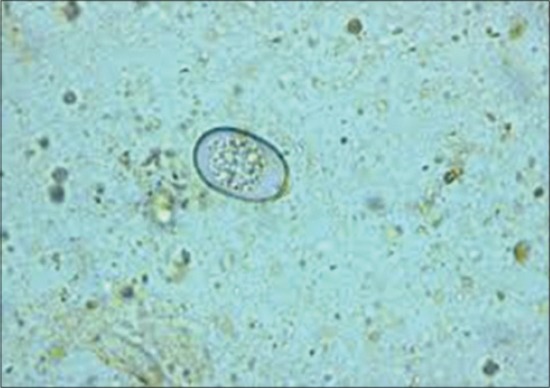
*Eimeria* oocyst as seen under a microscope (Sporozoite inside) (×100).

**Figure-3:**
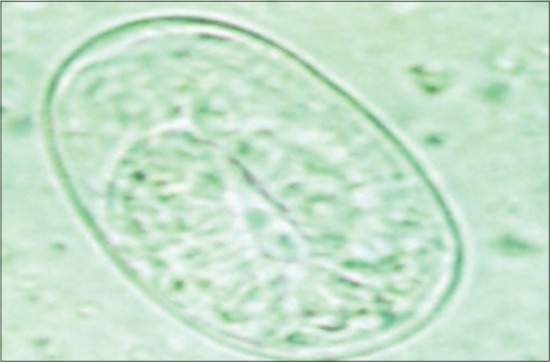
*Strongyloides papillosus* egg with larva inside (×400).

There were 110 calves enrolled in the study (48 females and 62 males), with a total of 220 fecal samples collected from the 109 farms (including the farm with twins). The results of the fecal examinations revealed *Eimeria* and *Cryptosporidia* as the protozoal infections infecting the calves, and *Strongyloides* as the helminth infection present among the study calves. There were no *Giardia* cysts detected in the fecal samples of the calves. Figure-1 shows a *Cryptosporidia* oocyst staining pink against a green malachite background. Figures-2 and -3 show an *Eimeria* oocyst and a *Strongyloides papillosus* egg as they appeared under a light microscope, respectively.

Table-1 shows the prevalence of *Eimeria, Cryptosporidia*, and *Strongyloides* infections among the study calves. There were no *Giardia* cysts detected in the fecal samples of the calves. Among the three parasites, *Eimeria* had the highest prevalence and *Strongyloides* was the least prevalent. Overall, a total of 52.7% (58/110) of the calves were infected with at least one of the parasites for at least one test sample, with 37.3% (41/110) infected at 4 weeks and 28.2% (31/110) infected at 6 weeks. The prevalence of *Eimeria, Cryptosporidia*, and *Strongyloides* was numerically but not statistically significantly higher at 4 weeks than at 6 weeks of age (p-value = 0.64; 0.47; 0.11, respectively). Moreover, the confidence intervals for the two ages of calves were overlapping for the three parasites. Only 7.3% (8/109), 0.9% (1/107), and 0.9% (1/109) of the study calves were infected at both 4 and 6 weeks of age with *Eimeria*, *Cryptosporidia*, and *Strongyloides*, respectively.

**Table-1:** Prevalences of *Eimeria, Cryptosporidia*, and *Strongyloides* in 110 calves reared in 109 smallholder dairy farms in Mukurwe-ini District, Kenya, 2013.

Parasitic condition	Prevalence							

	4 weeks		6 weeks		At 4 and 6 weeks		At 6 or 4 weeks	
			
	%	95% CI	%	95% CI	%	95% CI	%	95% CI
*Eimeria*	30.0 (33/110)	(21.8-39.6)	20.2 (22/109)	(13.3-29.2)	7.3 (8/109)	(3.5-14.4)	42.7 (47/110)	(33.5-52.5)
*Cryptosporidia*	8.2 (9/110)	(4.0-15.4)	6.5% (7/107)	(2.9-13.5)	0.9 (1/107)	(0.1-5.8)	13.6 (15/110)	(8.1-21.8)
*Strongyloides*	3.7 (4/109)	(1.2-9.7)	2.7% (3/110)	(0.7-8.4)	0.9 (1/109)	(0.1-5.8)	5.4 (6/110)	(2.2-12.0)

CI: Confidence interval

Mixed parasitic infections were reported in 10.3% (6/58) and were only detected in 4 week old calves; *Eimeria* and *Cryptosporidia* 5.2% (3/58), *Eimeria* and *Strongyloides* 3.4% (2/58), *Eimeria, Cryptosporidia*, and *Strongyloides* 1.7% (1/58). All mixed infections occurred with *Eimeria* and one or more of the other parasites.

## Discussion

Our study showed the presence of *Eimeria* oocysts, *Cryptosporidia* oocysts, and *Strongyloides* eggs in 4 and 6 weeks old dairy calves. Their prevalences were higher at 4 weeks of age compared to 6 weeks of age for all parasites. Calves are born immunologically naïve (immature immunity) [48] and their immunity matures with time, therefore calves at 6 weeks of age are able to mount a higher primary immune response than at 4 weeks of age [49]. Therefore, the development of some immunity could have partially led to the slightly lower prevalences at 6 weeks. There was, however, no significant difference in the prevalence of the parasitic conditions during the two time periods. The calves at both 4 and 6 weeks are still pre-weaned, hence mainly feeding on milk and small amounts of forage and calf pellets. Therefore, the sources of infection were likely similar. Mixed infections were only reported at 4 weeks, possibly due to the active immunity that is more pronounced at 6 weeks, hence reducing the number of overall infections and thus mixed infections as well.

*Cryptosporidium* species were detected in the study calves and the common species affecting calves have been known to have zoonotic potential [50,51]. Young calves are therefore a source of infection to human beings [13-15], especially in smallholder systems where hand-feeding is common [52]. Disadvantaged communities, such as rural areas where poverty levels are high, experience a greater impact of cryptosporidiosis which commonly has co-infections with other GI parasites [12,53].

Eimeriosis has been reported to be a common disease of calves [8,54-56], especially if they are kept in confinement [54,57]. Our study shows the overall infection prevalence of the calves at 4 or 6 weeks by *Eimeria* was 42.7%. This prevalence is lower than previous reports in Kenya of 67.4% by [58] and other reports in Ethiopia, [54] (68.1%) and (47.1%) in China [25].

Conversely, our prevalence was higher than reports in other regions: 19.8% in Zimbabwe [45], 22.7% [59] and 31.9% [60] in Ethiopia; 22.5% in Turkey [61], and 27.2% in Pakistan [57]. The wide prevalence variations may be associated with the differences in agroecology, farm management and husbandry in the different countries, the epidemiological status of *Eimeria* on the study farms and/or diagnostic tests used in the areas. Although *Eimeria* parasites were isolated from our study animals, there was no clinical disease observed on the biweekly visits. This finding suggests that most *Eimeria* infections in these calves were mild or sub-clinical and were more likely important in causing negative calf performance than clinical disease [57]. *Eimeria* oocysts were common among the calves, because of the ubiquitous nature of the oocysts on many farms [62]. Most farmers in the study area keep calves in confinement, and this has been reported to cause build-up of the hardy environmental oocysts [56], especially if hygiene is poor, hence increasing infection [59].

*Cryptosporidium* species have been reported in several species of young animals including bovine calves [63]. Transmission of the parasite is by the fecal-oral route, and the disease is readily transmissible since the oocysts persist for long periods in suitable environments [64], and very low numbers are required for infection to occur [65]. There was evidence that young calves (up to 6 weeks) from Mukurwe-ini District were infected by *Cryptosporidia*, and there is a need for strict adherence to good husbandry practices and hygiene. The overall prevalence of *Cryptosporidia* in the study calves during the study period was 13.6%. The prevalence was similar to other reports of 18% in Kenya [17], 13.2% in Turkey [61], and 17.1% in Argentina [63], but higher than the 3.1% found in beef calves in Canada [66].

Our *Cryptosporidium* prevalence was however lower than reports in other parts of Africa, although the studies in Africa are limited: 27% in Tanzania [11]; 38% in Uganda [67], 26.1% in Algeria [68], and 86.7% in Tunisia [69]. Other parts of the world have also reported a higher prevalence than the one reported in our study: 27% in Mexico [70], and 27% and 30% in Ontario, Canada [71,72], possibly due to the use of highly sensitive techniques such as ELISA and polymerase chain reaction. The apparent variability of prevalence between geographical localities and reports may reflect differences in the levels of calf management practices employed at the farm level, housing-related factors (i.e., single housed calves, cleanliness of the calf sleeping places), calf-related factors at the time of sampling (diarrheic versus non-diarrheic), nature of the study (cross-sectional versus longitudinal studies), and fecal screening technique used [73]. The prevalence was higher at 1 month than at 1½ months, and this agrees with other findings [74-76] that *Cryptosporidium* is common in calves up to 1 month of age.

*Strongyloides* was the main helminth infection reported in our calves up to 6 weeks of age. It has been detected as early as 2 weeks of age [77,78], and this has been associated with the immature immunity of calves. In our study, the prevalence of *Strongyloides papillosus* at 4 and/or 6 weeks of age was 5.4%, however, there were no strongyle eggs found. Our *Strongyloides* prevalence was lower than that reported (34.5%) in Serbia [77], and in Mali (39%) [48], but higher than the 2.0% in Zimbabwe [45], 4.0% in Kenya [8], and 4.3% in Czech Republic [36]. Contrary to *Strongyloides*, strongyles have been reported in calves more than 4 months of age [77] since at this age, there is increased forage ingestion, hence greater likelihood of ingesting the infective stages of the parasite. *Strongyloides papillosus* seems to infect mainly calves with its excretion diminishing by 6-8 months of age [45,79]. Excretion of *Strongyloides* eggs has been reported to be positively correlated to excretion by the dam, which is linked to vertical transmission of this parasite [48].

A relatively high prevalence of *Giardia* cysts has been detected in dairy calves elsewhere, with the cysts being detected in dairy calves as early as 1 month of age [30,74,80,81]. However, in our study, there were no *Giardia* cysts detected. This is contrary to other studies that reported existence of this protozoan, especially in dairy calves of similar age as the study calves; 14.7% [68] in Algeria; calves of 5-week-old, a prevalence of 44% in Myanmar was reported [30]; 85% in beef calves in the USA [82], and an overall prevalence of 32.5% in 6-week-old calves in Azerbaijan [83]. *Giardia* tends to be a chronic infection in calves; hence calves older than 8 weeks continue to be infected [72]. It is speculated that this protozoan may be absent in SDF in the study area since no previous literature has documented its prevalence in the study area. The absence of *Giardia* in calves may also be because of the limited water intake of the study calves in this area, and/or the water may not have been contaminated with *Giardia* because many farmers used water from wells or rainwater from cisterns.

## Conclusions and Recommendations

This study has shown that *Eimeria, Cryptosporidia*, and *Strongyloides* in calves are prevalent among SDF in the study area; hence the importance to place control measures against these conditions as early as 1 month of age. Although the overall prevalence, and a number of multiple and mixed infections appeared to be higher at 4 weeks than 6 weeks, this difference was not statistically significant. *Eimeria* infection in calves seems to have a higher prevalence than the other parasites, hence the need to improve farm husbandry practices to reduce exposure to infective oocysts.

*Cryptosporidium* infection of calves within the first 2 months of age has been reported to be by *C. parvum* which is zoonotic [84]. There is, therefore, a need to characterize the *Cryptosporidium* species in the study area so that the zoonotic potential can be assessed. Additionally, more sensitive techniques (PCR or ELISA) need to be used for detection of *Cryptosporidium* species in these calves to reduce false negative results. Characterization of the *Eimeria* species to assess the prevalence of pathogenic species (*E*. *bovis* and *E*. *zuernii*) among the calves present in our study area would also be helpful. Further investigations of *Giardia* cysts, using a larger sample size of calves that are older needs to be undertaken.

## Authors’ Contributions

GS, SR, and JV conducted the field data collection. GS and GKG wrote the draft manuscript. All authors were involved in the preparation of data collection materials, and the revision and approval of the final manuscript. SR, JV, GKG, and JW were involved in funding acquisition. GS, SR, JV, GKG, JW, FU, CMM, and OM were all involved in formulating the study design and methods of implementation of the study.
